# Rethinking immunization programs through the life course approach

**DOI:** 10.3389/fpubh.2024.1355384

**Published:** 2024-02-29

**Authors:** Evelyn Balsells, Margherita Ghiselli, Carolina Hommes, Beatriz Nascimento Lins de Oliveira, Ana Lucía Rosado-Valenzuela, Enrique Vega

**Affiliations:** Pan American Health Organization, Washington, DC, United States

**Keywords:** life-course, vaccination, immunization, immunosenescence, IA2030

## Abstract

The world continues to undergo a profound demographic shift toward increasing longevity –but quality of life is not improving correspondingly. At the same time, countries are taking stock of the negative impacts of the COVID-19 pandemic on national immunization programs. The pandemic exacerbated the declines in vaccination coverage for multiple vaccine-preventable diseases (VPD). To ensure that all persons receive all the vaccines for which they are eligible, it is time to consider how applying a life course approach (LCA) to immunization programs can help reinvigorate and redesign actions for greater vaccine uptake. In this mini review, we present the key concepts and principles of the LCA as applied to national immunization programs. Also, we offer recommendations on how health systems can achieve regional and national goals to ensure all people receive the recommended vaccine doses at every stage of life, thus ensuring the greatest benefits for individuals and societies.

## Introduction

As longevity continues to increase, it presents opportunities and challenges for immunization efforts. In the Americas, life expectancy (LE) has increased by approximately 15 years in the past half century (1). Yet, the average number of years a person lives in “full health” (healthy life expectancy – HALE) (2) is 66, compared to an average lifespan of 77 years (3). Immunization strategies such as vaccination, breastfeeding and immunotherapies can contribute to the attainment of a full health status at each stage of life.

Established in 1974, the Expanded Program on Immunization (EPI) has contributed to the prevention of millions of deaths, especially among children younger than five years (4). Yet over the last decade, the Americas have seen a noticeable decline in vaccination coverage rates for all antigens (5). The advent of the COVID-19 pandemic further exacerbated this trend, leaving 1.3 million children younger than one year without a single dose of vaccine in 2022. Nonetheless, the pandemic also presented an opportunity for the Americas to strengthen and expand vaccination platforms beyond childhood – reaching large portions of adolescents, adults, older adults and pregnant women with the COVID-19 vaccines (6). This expansion of national immunization programs is in line with the recommendations of the Immunization Agenda 2030 (IA2030), which recognizes that the LCA framework is necessary to maximize the impact of immunization across an individual’s life stages and generations (7). Yet, while some countries have made enormous progress in extending both COVID-19 and routine vaccination services beyond the pediatric population (5), many still engage with adolescents and adults only in the context of COVID-19 vaccination. These missed opportunities should be documented and addressed to allow all segments of the population to fully benefit from the immediate and long-term protection of vaccines (8).

## Study design

In November 2022, the Pan American Health Organization/World Health Organization (PAHO/WHO) held a webinar titled “Building a Better Immunity: A Pathway to Healthy Longevity” (9), to discuss the implications for the present and future of the life course approach in the fields of medicine and public health, as well as to better understand the existing applications of this approach to immunization programs in the region of the Americas and in the world. The webinar included presentations from academic researchers and WHO officers to describe the principles of the LCA to the Ministries of Health of the countries and territories of the Americas, as well as propose first steps to integrate these concepts into the strategic plans of national immunization programs.

The proceedings from the webinar were formatted and expanded to develop a PAHO technical document of the same title (10). For each LCA principle, we searched the PAHO and WHO literature for instances of vaccines that are already included in national immunization schedules and exemplify the application of LCA concepts in national vaccination policies or strategies. Also, we searched the scientific literature to include the biological mechanisms that explain the reasons for favoring the application of specific vaccines at specific ages or under specific conditions. Finally, we provided examples of how the LCA can be used to promote vaccination services as a component of an integrated primary healthcare system – where members of each age group may be offered a complete package of complementary services to boost their overall health and wellbeing depending on their specific needs.

This publication summarizes the key points of the PAHO technical document and adapts them for further dissemination to Ministries of Health outside the Americas, as well as to the broader scientific community.

## LCA principles applied to health

The LCA can apply eight principles to a health framework (11) to maximize the potential of improving health outcomes and extend healthy lives. First, health develops over dynamic trajectories that are impacted by transitions –moments in life when there is loss or gain of health functions– and the cumulative impact of positive and negative exposures throughout life. Also, each trajectory can be adjusted during critical periods –moments where health interventions can be applied to generate the strongest and most permanent health responses. LCA concepts also contemplate external forces that impact health. The historical and societal context where individuals and communities live –temporality– and the human agency to make individual decisions are key drivers of a person’s health status throughout life. Finally, the interconnectivity between generations ensures the transfer of traits and resources, including health status. Also, the health status of other people who live in the same environment –linked lives– can affect the health of individuals and families.

## Why a life course approach to immunization

The LCA considers health as an evolving capacity that develops dynamically over a person’s life, across generations and within a community (12, 13). As individuals age, their immune system changes its capacity to respond to pathogens, and eventually declines through a process called immunosenescence (14). Also, individuals experience inflammaging, an increase in pro-inflammatory markers (15) that can disrupt health capacities when the body is unable to mount a response to specific pathogens. To respond to these dynamic trajectories, the LCA promotes person-centered interventions to generate health benefits beyond survival. An example are the national immunization programs that improve individuals’ capacity to fight the consequences of infections and therefore generate lasting health for persons and their community. By taking note of the moments when the immune system is weakest or in transition, vaccination schedules can be adjusted to deliver vaccine doses that enhance a person’s protection against those vaccine preventable diseases (VPD) to which it is most vulnerable at each stage of life (9). When administered to a sufficient number of vulnerable persons, vaccination programs can reduce the morbidity and mortality burden of an entire community and future generations.

## Life course approach principles applied to vaccination

The LCA principles can be applied to national immunization programs (15), so they are optimally designed to take advantage of critical periods, improve health trajectories and minimize the effects of immunosenescence. On a broader scale, the LCA can inform the development of vaccination platforms that take note of the critical periods in a person’s life to: (a) design interactions with vaccination services; (b) generate vaccine demand by coupling this service with other age-appropriate health practices; and (c) develop information systems that track a person’s medical history (including vaccinations) and identify the most appropriate moments for intervention. Depending on the level of intervention selected by the country, LCA principles can be applied to the national immunization program to benefit individuals, generations, and communities (15).

## Individual life trajectories and immunization

Vaccines were developed for different age groups to help maintain the plasticity of innate and adaptive immune systems to respond to external stimuli (infection or vaccination). Therefore, primary schedule vaccine doses are designed to be administered at critical periods of a life trajectory and at moments of transition between life stages to help optimize the body’s response to the pathogens that most affect a specific age group. It is recommended these periods are extended through catch-up vaccination (8). Catch-up vaccine doses ensure that the whole length of the critical period is used to provoke a response or prevent illness that can last into other life stages. As we age, immunosenescence has a cumulative impact on a person’s inability to respond to infections and affects all individual life trajectories. To compensate, public health officials need to adjust national vaccination schedules to include booster doses at the time when antibody titers against a specific antigen are known to have declined.

These considerations are especially important for high-risk individuals who experience the cumulative impact of risk factors through repeated exposures to the pathogen (e.g., health workers) or chronic health conditions (e.g., immunocompromised persons). Also, persons living in situation of vulnerability (e.g., migrants, indigenous groups, refugees, internally displaced populations, people in closed institutions) are at higher risk of developing disease due to the unfavorable social determinants that affect their daily lives. For them, the immunity generated through vaccination reduces the risk of disease infection, as well as the interaction of disease sequelae with other comorbidities later in life.

In the Americas, some examples of how vaccines can impact a person’s life trajectory and produce long-lasting health benefits are:

- Administration of Bacille Calmette-Guérin (BCG) and hepatitis B vaccines within the first 24 h of life to protect newborns from tuberculosis (TB), in line with PAHO recommendations (16). This is a critical period in the life of a newborn because the immune system is still developing and is especially vulnerable to this disease. If high coverage rates of BCG vaccination within the first 24 h cannot be achieved consistently, a country may consider introducing a catch-up policy to allow this dose to be administered during the first year of life – in line with WHO recommendations (17) – to boost population immunity.
- Administration of the vaccine against the Human Papilloma Virus (HPV) to adolescent girls. One dose administered prior to sexual intercourse minimizes the risk of developing precancerous and cancerous lesions later in life (18).
- Administration of 6 doses (3 primary plus 3 boosters) of tetanus toxoid-containing vaccines (TTCV) throughout childhood and adolescence. Since immunity against tetanus wanes over time, it is recommended that adults receive a booster dose every 10 years to ensure protection throughout life (19).

## Intergenerational impact

The LCA pays attention to the intergenerational benefits of vaccination programs, which includes the transmission of maternal antibodies to the fetus via vaccines and to the newborn via breastmilk. Both these instances are clear example of linked lives and the transfer of traits and resources. Also, these are cost-effective strategies that optimize health outcomes across two generations (16, 20). National immunization programs should ensure that: (a) vaccines are available to pregnant women; and (b) they receive all doses for which they are eligible.

Another application of the principle of transfer of traits and resources is the sharing of habits and behaviors within a social unit (e.g., the family nucleus). These behaviors may be favorable or unfavorable to good health. For example, research from countries in the Americas has shown that parents of unvaccinated children are more likely to not trust COVID-19 vaccines than parents of vaccinated children (20). Therefore, national immunization programs could offer outreach services and demand generation activities that target multiple members of a social unit at once, so that vaccine uptake by one member may trigger greater acceptance within the unit. PAHO’s own Vaccination Week in the Americas (21) is an example of an outreach event that targets all age groups.

## Human agency and temporality, and their impact on community health

The LCA principles can be applied to improve health at the community level. Through human agency, persons who are at high-risk of contracting a VPD or of being hospitalized or dying from it can choose to receive a vaccine dose. By lowering the morbidity and mortality rate in population groups, the burden of disease of the entire community is lowered as well –since high-risk groups are the greatest contributors to that same burden of disease. Also, the LCA highlights temporality trends that shape community patterns in health-seeking behaviors. Specifically, social, cultural, and historical factors at a given point in time can impact vaccine acceptance and uptake. Therefore, human agency can be shaped by temporal trends, and this agency can vary between age groups and risk groups. The combination of these factors can affect the vaccination status in a particular life stage.

Public health officers can influence human agency among high-risk persons though targeted outreach efforts and communications campaigns – thus improving the health of the community. These operations must take into account the temporal issues so that vaccination communication campaigns consider potential habits and behaviors of the target population, the social determinants that affect one’s abilities to make decisions about his/her health, and the context in which people live. Thus supported, individuals can better shape their actions to support healthy life trajectories – to the benefit of the community as a whole.

A recent example of a LCA application to national vaccination strategies is the prioritization of COVID-19 vaccines to high-risk groups during the pandemic (22, 23). This decision maximized the impact of the vaccines on the disease burden of the populations by preventing severe disease, hospitalization, and death among the most vulnerable members of society, and relieved health systems of additional burden of care (24, 25).

## Toward implementation of the life course approach to immunization

While there is growing evidence in the scientific literature on the benefits of applying LCA principles to vaccination programs, the implementation of these principles to national immunization programs lags behind. Yet there is a need to rethink immunization programs to minimize missed opportunities and extract the full benefit of vaccines for persons of all ages. To develop strong vaccination platforms and immunization strategies, systemic changes are necessary (26). We propose that these changes focus on six elements of a national health system.

1. *Advocacy*: Strong advocacy to implement the principles of the LCA to national immunization programs must rest with high-level officials and policymakers. This advocacy should translate into expanded vaccination services that reach all persons and offer vaccine doses to all eligible persons according to their age.
2. *Financing*: To sustain expanded vaccination platforms that promote vaccine doses beyond the pediatric population, adequate and consistent financial resources are required. Funds should translate to the engagement of additional human resources to manage the increased workload, as well as training sessions to ensure that all vaccine doses are adequately administered and recorded in the vaccination registers. Such resources should be seen as a long-term investment given the economic and social benefit of immunizations (i.e., reduced healthcare costs from having prevented infectious diseases and chronic conditions; longer and healthier lifespans for a larger portion of the population).
3. *Integration of person-centered services*: The consistent expansion of vaccination services to all age groups – including the administration of booster and catch-up doses – requires synergy with other essential health services that are person-centered, age-appropriate and designed around the needs and environments of eligible persons.
4. *Human Resources*: The positive impact of the LCA on national immunization programs must be communicated broadly to public health officials, health administrators, health providers and vaccinators. Additional training resources must be provided to ensure that vaccines are promoted to individuals of each age group whenever they come in contact with essential services.
5. *Information and evidence generation*: The benefits of the LCA for national immunization programs must continue to be researched, tracked, and documented to assess how these changes to vaccination services are impacting broader population health outcomes, healthcare, and public health priorities. Health systems should invest in longitudinal research and information systems (e.g., electronic immunization registries) to monitor improvements in limiting missed opportunities and measuring benefits beyond the prevention of a single VPD.
6. *Equity*: To maximize the benefits of vaccines, they should be available to everyone, everywhere. Countries should establish targeted strategies to reach vulnerable populations. Outreach operations and cultural dialogs are well-documented activities that help reduce inequalities in vaccination coverage rates across the life course.

## Conclusion

Rethinking immunization programs from the perspective of a LCA presents an opportunity for adjusting vaccination services to obtain greater health benefits at the individual and community level. An LCA-centered immunization program addresses the health needs of individuals over time by considering their life trajectories and critical periods. Also, it can broaden its scope by designing its interventions to improve the health of communities for generations to come. At least six essential elements of the health system should be targeted when integrating the LCA principles to national immunization programs.

## Author contributions

EB: Writing – original draft, Writing – review & editing. MG: Conceptualization, Methodology, Project administration, Supervision, Writing – original draft, Writing – review & editing. CH: Conceptualization, Writing – original draft, Writing – review & editing. BN: Writing – review & editing. AR-V: Conceptualization, Writing – original draft, Writing – review & editing. EV: Conceptualization, Supervision, Writing – review & editing.

## References

[1] United Nations. (2022). Revision of world population prospects. 2022. Available at: https://population.un.org/wpp/ (Accessed May 8, 2023)

[2] WHO. (2023). The Global Health Observatory – healthy life expectancy (HALE) at birth. Available at: https://www.who.int/data/gho/indicator-metadata-registry/imr-details/66 (Accessed May 8, 2023)

[3] WHO. (2023). Life expectancy and healthy life expectancy – data by WHO region. Available at: https://apps.who.int/gho/data/view.main.SDG2016LEXREGv?lang=en (Accessed May 8, 2023)

[4] Etienne CF. Expanded program on immunization in the Americas: 40 years. Rev Panam Salud Pública. (2017) 41:1–2. doi: 10.26633/RPSP.2017.139, PMID: 31384266 PMC6645206

[5] WHO/UNICEF. (2022). WHO/UNICEF estimates of National Immunization Coverage (WUENIC). Available at: https://www.who.int/teams/immunization-vaccines-and-biologicals/immunization-analysis-and-insights/global-monitoring/immunization-coverage/who-unicef-estimates-of-national-immunization-coverage (Accessed January 2, 2024)

[6] PAHO. (2021). COVID-19 vaccination in the Americas. Available at: https://ais.paho.org/imm/IM_DosisAdmin-Vacunacion.asp (Accessed January 2, 2024)

[7] WHO. (2020). Immunization agenda 2030- SP4: Life course and integration. Available at: https://www.immunizationagenda2030.org/strategic-priorities/life-course-integration (Accessed May 8, 2023)

[8] Tampi M Carrasco-Labra A O’Brien KK Velandia-González M Brignardello-Petersen R. Systematic review on reducing missed opportunities for vaccinations in Latin America. Rev Panam Salud Pública. (2022) 46:1. doi: 10.26633/RPSP.2022.65, PMID: 35747470 PMC9211032

[9] PAHO. (2022). Building a better immunity: A pathway to healthy longevity. Available at: https://www.paho.org/en/events/building-better-immunity-pathway-healthy-longevity (Accessed May 8, 2023)

[10] PAHO. (2021). Building health throughout the life course. Concepts, implications, and application in public health. Available at: https://iris.paho.org/handle/10665.2/53409 (Accessed May 8, 2023)

[11] Hommes C Ambrose A Vega E Martinez R. Four reasons for adopting a life course approach to health in the COVID-19 era and beyond. Rev Panam Salud Pública. (2022) 46:1. doi: 10.26633/RPSP.2022.182, PMID: 36406294 PMC9668044

[12] PAHO. (2023). Healthy life course. Available at: https://www.paho.org/en/topics/healthy-life-course (Accessed May 8, 2023)

[13] Goronzy JJ Weyand CM. Understanding immunosenescence to improve responses to vaccines. Nat Immunol. (2013) 14:428–36. doi: 10.1038/ni.2588, PMID: 23598398 PMC4183346

[14] Santoro A Bientinesi E Monti D. Immunosenescence and inflammaging in the aging process: age-related diseases or longevity? Ageing Res Rev. (2021) 71:101422. doi: 10.1016/j.arr.2021.101422 34391943

[15] Philip RK Attwell K Breuer T Di Pasquale A Lopalco PL. Life-course immunization as a gateway to health. Expert Rev Vaccines. (2018) 17:851–64. doi: 10.1080/14760584.2018.1527690, PMID: 30350731

[16] PAHO. (2018). Maternal and neonatal immunization field guide for Latin America and the Caribbean. PAHO. Available at: https://iris.paho.org/handle/10665.2/34150 (Accessed May 8, 2023)

[17] WHO. (2018). BCG vaccines: WHO position paper – February 2018. Available at: https://www.who.int/publications/i/item/who-wer9308-73-96 (Accessed January 2, 2024)

[18] Lei J Ploner A Elfström KM Wang J Roth A Fang F. HPV vaccination and the risk of invasive cervical Cancer. N Engl J Med. (2020) 383:1340–8. doi: 10.1056/NEJMoa1917338 32997908

[19] PAHO. (2018). Third ad hoc meeting of the technical advisory group (TAG) on vaccine-preventable diseases – final report. https://www.paho.org/en/documents/03-ad-hoc-tag-final-report-2018 (Accessed January 2, 2024)

[20] Nguyen KH Nguyen K Mansfield K Allen JD Corlin L. Child and adolescent COVID-19 vaccination status and reasons for non-vaccination by parental vaccination status. Public Health. (2022) 209:82–9. doi: 10.1016/j.puhe.2022.06.002, PMID: 35870290 PMC9189141

[21] PAHO. (2023). Vaccination week in the Americas 2023. https://www.paho.org/en/campaigns/vaccination-week-americas-2023 (Accessed May 8, 2023)

[22] WHO. (2020). WHO SAGE values framework for the allocation and prioritization of COVID-19 vaccination. Available at: https://apps.who.int/iris/bitstream/handle/10665/334299/WHO-2019-nCoV-SAGE_Framework-Allocation_and_prioritization-2020.1-eng.pdf

[23] WHO. (2020). WHO SAGE roadmap for prioritizing uses of COVID-19 vaccines. Available at: https://www.who.int/publications/i/item/WHO-2019-nCoV-Vaccines-SAGE-Prioritization-2022.1 (Accessed May 8, 2023)

[24] McNamara LA Wiegand RE Burke RM Sharma AJ Sheppard M Adjemian J. Estimating the early impact of the US COVID-19 vaccination programme on COVID-19 cases, emergency department visits, hospital admissions, and deaths among adults aged 65 years and older: an ecological analysis of national surveillance data. Lancet. (2022) 399:152–60. doi: 10.1016/S0140-6736(21)02226-1, PMID: 34741818 PMC8565933

[25] Rojas-Botero ML Fernández-Niño JA Arregocés-Castillo L Ruiz-Gómez F. Estimated number of deaths directly avoided because of COVID-19 vaccination among older adults in Colombia in 2021: an ecological, longitudinal observational study. F1000Research. (2022) 11:198. doi: 10.12688/f1000research.109331.2 35811799 PMC9214267

[26] Aguado T Goodwin J Holt D. (2018). A life-course approach to vaccination: Adapting European policies. Available at: https://www.healthpolicypartnership.com/app/uploads/A-life-course-approach-to-vaccination-adapting-European-policies.pdf (Accessed May 8, 2023)

