# Corrigendum: Rethinking immunization programs through the life course approach

**DOI:** 10.3389/fpubh.2024.1416627

**Published:** 2024-05-07

**Authors:** Evelyn Balsells, Margherita Ghiselli, Carolina Hommes, Beatriz Nascimento Lins de Oliveira, Ana Lucía Rosado-Valenzuela, Enrique Vega

**Affiliations:** Pan American Health Organization, Washington, DC, United States

**Keywords:** life-course, vaccination, immunization, immunosenescence, IA2030

In the published article, there was an error in the Copyright statement.

The correct statement appears below:

“Copyright © Pan American Health Organization 2024. Licensee Frontiers Media SA. This is an open access article distributed under the terms of the Creative Commons Attribution IGO License (http://creativecommons.org/licenses/by/3.0/igo/legalcode), which permits unrestricted use, adaptation (including derivative works), distribution, and reproduction in any medium, provided the original work is properly cited. In any reproduction or adaptation of this article there should not be any suggestion that PAHO or this article endorse any specific organization or products. The use of the PAHO logo is not permitted. This notice should be preserved along with the article's original URL.”

An Author's Disclaimer was missing from the original article. The correct Author's Disclaimer is as follows:

“The authors are staff members of the Pan American Health Organization. The authors alone are responsible for the views expressed in this publication and they do not necessarily represent the views, decisions or policies of the Pan American Health Organization.”

The authors apologize for this error and state that this does not change the scientific conclusions of the article in any way. The original article has been updated.

